# What role will physiological resilience play in brown-white fat dynamic in obesity management?

**DOI:** 10.3389/fphar.2025.1691149

**Published:** 2026-01-05

**Authors:** Oksana Zayachkivska, Iryna Kovalchuk, Iryna Muzyka, Maryana Savytska

**Affiliations:** 1 American University of Health Sciences, Signal Hill, CA, United States; 2 Danylo Halytsky Lviv National Medical University, Lviv, Ukraine; 3 Novoyavorivsk Hospital Named After Yurii Lypa, Lviv, Ukraine

**Keywords:** physiological resilience, adipocentric model, stress, metabolic health, fat, brown adipocyte, white adipocyte, obesity management

## Introduction

The current obesity prevalence presents a significant paradox: despite notable therapeutic advances, including widespread use of GLP-1 receptor agonists (GLP-1RA) and SGLT2 inhibitors (SGLT2i) valued at $46.7 billion globally, the prevalence is expected to rise. Projections for 2035 estimate economic costs of $4 trillion (Author Anonymous, 2018; Dellgren et al., 2024). These medical and socioeconomic challenges, linked to increasing obesity-related cardiovascular and neurodegenerative comorbidities, highlight fundamental limitations in current pharmacotherapeutic approaches (Coral et al., 2025; Pede et al., 2025; Hall, 2025; Zeng et al., 2025). However, the recently introduced adipocentric model for understanding metabolic health and preventing metabolic alterations offers a promising way forward. Over 75 medical societies recognize that the dynamics of brown and white fat (adipose tissue) are key components of the definition and diagnosis of obesity (Rubino et al., 2025). Today, obesity is an umbrella term encompassing a big group of diseases/with metabolic disorders (Supplementary Material 1). Therefore, the new diagnostic approach based on the adipocentric model for prevention and early detection of pre-obesity and clinical obesity will help identify body changes that are predictors or signs of metabolic alterations. This promising strategy could slow the emergence of a new pandemic of metabolic diseases and related comorbidities.

At the same time, a contemporary understanding of metabolic health through the lens of physiological resilience reveals how personalized responses to external stressors determine health outcomes and can help implement precise preventive interventions. Hans Selye, the “father of biological stress,” was an academic leader who first introduced the concept of “stressors” through his pioneering observations on animal models as well as on clinical findings on sudden increases in gastroduodenal ulcer hospitalizations during Nazi rocket attacks and air raids in London (1942–1943) and involvement of brown fat in stress response (Szabo et al., 2012). Regarding the WHO report, during the outgoing war in Ukraine, there was an increase in type 2 diabetes mellitus twice, and three-fifths of adults aged 18–69 years in Ukraine were overweight and obese based on BMI (18.5–24.9 kg/m2) (WHO, 2023). These evidence-based facts reveal that chronic stress induces metabolic diseases.

Selye’s three-stage stress response involving hypothalamic-pituitary-adrenal (HPA) axis regulation includes alarm, resistance, and exhaustion. His later theory of “cross-resistance” shows that organisms exposed to mild stressors tend to perform better when faced with more intense versions of the same stressor (Selye, 1952; Selye, 1956). These findings, along with subsequent evidence on autonomic nervous system (ANS) balance, as reflected in heart rate variability (HRV) indices, and inflammatory tone, are essential for understanding the relationship between physiological resilience and metabolic changes driven by brown-white adipose tissue dynamics (Williams et al., 2019; Ramiro-Cortijo et al., 2025).

### Power of physiological resilience: Focus on brow-white fat plasticity

**FIGURE 1:**
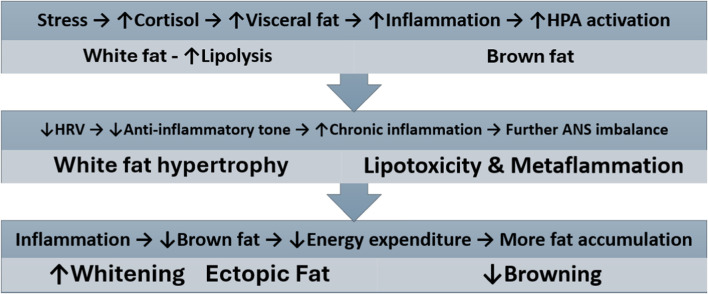
Physiological resilience–adiposity axis with multisystem feedback loops contributing to obesity pathogenesis. Chronic stress activates the hypothalamic-pituitary-adrenal (HPA) axis, increasing cortisol and visceral fat accumulation, which triggers inflammatory cascades. Reduced autonomic nervous system (ANS) flexibility and decline of physiological resilience (↓HRV) diminishes anti-inflammatory capacity, leading to white fat hypertrophy, lipotoxicity, and metabolic-associated inflammation (Metaflammation). Progressive inflammation impairs brown fat function and promotes whitening, reducing energy expenditure and creating a self-reinforcing cycle of fat accumulation.

Physiological resilience, an individual’s natural ability to handle stress effectively, depends on various factors, including age, pre-existing ANS flexibility, health conditions, metabolic health, lifestyle choices such as physical activity, sleep quality, and nutrition, and adaptation to cold or heat exposure (Jones and Kirby, 2025). Higher physiological resilience raises the threshold at which stressors cause discomfort and damage, while lower resilience makes one more vulnerable to these stressors. Because personal metabolic health is crucial for building physiological resilience, it can help predict and prevent pre-obesity. The bidirectional transformation between energy-storing white fat cells and thermogenic brown/beige fat cells creates a mechanistic link between stress adaptation and metabolic health, forming a resilience-adiposity axis. Its activity refers to metabolic flexibility (the ability to switch between glucose and fat/ketones as fuel sources). Metabolic inflexibility leads to dysregulation of energy metabolism and immune system. These interconnected multisystem loops, or the *resilience-adiposity axis*, with brown-white fat plasticity and dynamics at their core, represent an adipocentric model for practical assessment and strategies to prevent and manage obesity based on resting and challenge-recovery HRV indices, cortisol awakening response, cold-pressor or cold-exposure recovery, perceived stress scale with test–retest reliability (Figure 1).

The dynamic interplay between brown and white fat phenotypes is a fundamental mechanism through which physiological resilience influences metabolic health (Berthoud et al., 2021; Cabot et al., 2023). The process of “browning” fat involves β-adrenergic signaling cascades that promote mitochondrial biogenesis and UCP1 expression, effectively converting energy-storing white adipocytes into energy-dissipating brown-like cells (Bartelt and Heeren, 2014; Chouchani and Kajimura, 2019). This brown-white fat dynamic may be characterized by ‘whitening’ of brown adipose tissue (progressive lipid droplet accumulation, white fat hypertrophy, mitochondrial dysfunction, and loss of thermogenic capacity), decreased cellular defense and development of metaflammation (Muzyka et al., 2023a; Revenko et al., 2021). It is often associated with metabolic inflexibility, which is linked to chronic stress, aging, or metabolic disease. These resilience-adiposity axis activities reflect a series of challenge-recovery-growth waves across the fat plasticity continuum, with strong wave responses encouraging sustained browning and metabolic health by biofeedback, while disrupted wave patterns accelerate pathological whitening and the development of obesity. Moreover, adipocentric metabolic “personalized signature” has gained renewed clinical relevance in severe COVID-19 cases, where initial cortisol elevation is followed by adrenal exhaustion and glucocorticoid synthesis failure, with synthetic corticosteroid intervention proving lifesaving in many patients (RECOVERY Collaborative Group, 2021). On the other hand, the personalized “fingerprint” of the resilience-adiposity axis, which integrates multiple systems (adipose, autonomic, and inflammatory), has been useful for understanding patients with long COVID, a new disease related to the malfunctioning of adaptive capacity mobilization (Muzyka et al., 2023b; Szabo, 2020; Szabo, 2023). Thus, we hypothesize that subtyping obesity with an adipocentric model based on resilience profiling can predict: 1) preclinical obesity, 2) treatment response before anti-obesity therapy begins, and 3) the effectiveness of GLP-1RA and SGLT2i.

### Adipocentric model with resilience profiling predicts obesity subtyping

Based on our understanding that the prediction of metabolic disorders and the early assessment of obesity subtypes lack consistent correlation with clinical outcomes, several potential solutions can be helpful. Thus, should be considered to add to advanced anthropometric measurements (Gebreyesus et al., 2024; Sriram et al., 2024; Alfadhli et al., 2017; World Health Orga nization, 2011), a complex digital biomarker score integrating during standardized cold challenges (Matsumura et al., 2025; Motzfeldt et al., 2025; van der Lans et al., 2013; van Marken Lichtenbelt et al., 2009) (Supplementary Material 2). When findings are analyzed using machine learning algorithms, they can detect brown-white fat dynamic dysfunction with higher accuracy in pre-obese individuals (BMI 25–29.9 kg/m^2^) before clinical obesity manifests. This predictive accuracy exceeds that of traditional biomarkers (fasting glucose, lipid panel, and inflammatory markers) used to diagnose metabolic disorders. This hypothesis directly addresses the need for early detection biomarkers that preceded clinical manifestations. For this reason, it needs to test its comparative predictive accuracy against traditional cardiometabolic risk scores (Framingham, ASCVD). Moreover, it can provide evidence-based, relevant arguments for brown fat-targeted interventions, such as cold exposure therapy and exercise protocols, as well as for the validity of standardized uptake value measurements in PET-CT imaging used to detect and quantify brown fat (Suppl. 3).

Since physiological resilience, an individual’s natural capacity to manage stress effectively, depends on various factors, including age, pre-existing health conditions, metabolic and immunological status, lifestyle choices, physical activity, sleep quality, nutrition, and acclimatization to cold or heat exposure, autonomic adaptability plays a central role for metabolic health (Herz et al., 2021; Matsushita et al., 2021; Agueda-Oyarzabal and Emanuelli, 2022; Hanssen et al., 2015; Sepa-Kishi et al., 2018). Dysregulation of the HPA axis leads to chronic cortisol elevation, which promotes stress-related obesity, activates ANS, the brain-gut axis, and increases inflammatory markers such as CRP, leading to several malfunctions in energy and glucose metabolism (Wachsmuth et al., 2022; Kataoka et al., 2014). Prolonged cortisol increase specifically promotes the buildup of visceral fat and boosts white adipose tissue lipolysis, releasing fatty acids. Glucocorticoids also directly affect fat distribution and can suppress brown fat activity, increase whitening and ectopic fat formation. The sympathetic nervous system is the principal stimulator of brown adipose tissue thermogenesis through norepinephrine release onto β3-adrenergic receptors. Brown fat activation is under direct control of central sympathetic circuits that respond to cold, metabolic signals, and thermoregulatory demands. Altered HRV reflects autonomic imbalance and is associated with increased inflammation and insulin resistance (Ramiro-Cortijo et al., 2025; Jones and Kirby, 2025). Findings about lower HRV, which correlates with elevated proinflammatory adipokines levels, and decreased vagal activity impairing anti-inflammatory mechanisms, help in understanding metabolic flexibility and its changes (Cypess et al., 2014).

### Adipocentric model with resilience profiling predicts therapeutic response

High physiological resilience elevates the threshold at which stressors cause discomfort and harm, whereas low resilience increases vulnerability to them. Since personal metabolic status is vital for developing physiological resilience, autonomic profiling and character of brown-white fat dynamics could be used to predict and prevent pre-obesity. Furthermore, it has potential for effective therapeutic interventions for clinical obesity (von Majewski et al., 2023; Nel et al., 2012; D et al., 2025).

Next, adipocentric model introduced for classified individuals by adipocentric criteria into distinct obesity subtypes: metabolically healthy obesity (MHO), metabolically unhealthy obesity (MUO), sarcopenic obesity, TOFI (Thin Outside, Fat Inside) phenotype, android versus gynoid distribution, or brown adipose tissue deficit obesity and further graded by physiological resilience capacity (assessed through autonomic adaptability metrics, stress tolerance testing, and challenge-recovery-growth wave analysis) will help predict therapeutic response. It will help select efficient nonpharmacological and pharmacological interventions for those who demonstrate significantly different therapeutic responses to matched versus mismatched interventions. Specifically, we hypothesize that based on the proposal adipocentric model with autonomic profiling, subtype-matched therapies (e.g., GLP-1 agonists for MUO, leucine supplementation with resistance training for sarcopenic obesity, cold exposure protocols for BAT deficit obesity) will help achieve greater reductions in cardiometabolic risk markers than standard (“one-size-fits-all approach”) at 12-month follow-up. In addition, the current failure of uniform treatment protocols across obesity subtypes suggests that an individualized approach is essential for long-term maintenance of metabolic improvements in the face of obesity heterogeneity. It is therefore the resilience-adiposity axis that is considered a promising adipocentric model for precise, individualized treatment protocols for obesity, matching interventions to autonomic tone, metabolic flexibility, and the inflammatory state.

## Discussion

Recent fundamental and clinical studies have altered the conceptual understanding of obesity, emphasizing the crucial role of brown-white fat dynamics. This dynamic, which represents the primary risk factor for developing clinical obesity (TOFI, MUO, MHO and other subtypes), is associated with both cardiovascular and neurodegenerative diseases. Three core pathophysiological features characterize the adipocentric model with reliance profiling: increased ectopic and visceral fat deposition, dysregulated adipokine secretion favoring pro-inflammatory states, and insulin resistance. The emerging ectopic and visceral fat accumulation are outcomes of brown-white dynamics. Together with novel physiology-based digital biomarkers, detection of brown-white fat dynamics with physiological resilience profiling, including, autonomic function tests and inflammation tone will be more beneficial than rather than static measurements. However, the causal directionality between resilience and brown fat dynamics remains uncertain. We need to clarify whether impaired resilience drives brown fat dysfunction or whether brown fat deterioration compromises resilience capacity. This bidirectional relationship complicates intervention design and the interpretation of outcomes. Even if the proposed hypotheses are validated, significant barriers to clinical implementation exist. Ultimately, precise approaches for obesity management could revolutionize prevention and treatment strategies. After evaluating the effectiveness of these interventions, the findings will help identify and analyze differences in brown-fat dynamics. The proposed adipocentric model with physiological resilience profiling focuses on dynamics and flexibility metabolic processes which integrated with other organ systems and reflect influence of external environmental factors (Table 1). It will likely enhance current understanding, moving beyond the simple distinction between obesity and pre-obesity to address the complex mechanisms underlying adipose tissue dysfunction. Moreover, it emphasizes the essential role of prevention through early biomarkers using HRV as an actionable and modifiable target for personalized both lifestyle and pharmacotherapy. Incorporating the proposed model of adipose tissue heterogeneity (brown-white dynamics) into pilot programs across diverse healthcare settings (academic medical centers, community health systems, primary care networks) with comparative effectiveness evaluation will lead to precise obesity medicine. It will open new opportunities to enhance the long-term outcomes of GLP-1RA and SGLT2i, tailored to individual resilience and adipose tissue phenotypes.

**TABLE 1:** Summary of the key evidence supporting adipocentric model with autonomic profiling and distinguishes established findings from testable hypothese requiring further investigation.

Domain	Established evidence	Testable hypothese
Brown fat plasticity	Cold exposure increases brown fat activity and thermogenesis; chronic stress induces brown fat whitening with mitochondrial dysfunction	Digital biomarkers combining optical, electrical, mechanical, and thermal modalities can detect ANS state and preclinical fat dysfunction before clinical obesity manifests
Obesity heterogeneity	MHO and MUO phenotypes show distinct metabolic profiles despite different BMI; visceral fat accumulation predicts cardiometabolic risk independent of total adiposity (sarcopenic obesity of TOFI)	Physiological resilience profiling can predict which MHO individuals will transition to MUO, enabling targeted preventive interventions before metabolic decompensation occurs
Stress-metabolism link	War-exposed populations show doubled T2DM incidence; COVID-19 patients with adrenal exhaustion require corticosteroid intervention; long COVID associates with resilience dysfunction	Repeated mild stressor exposure cold, physical exercise) can enhance physiological resilience, improving brown fat function and preventing stress-induced metabolic disorders
Therapeutic response	GLP-1RA show variable efficacy; sarcopenic obesity requires protein-enriched interventions; TOFI phenotype responds to exercise over pharmacotherapy	Adipocentric subtyping combined with resilience assessment can predict therapeutic responsiveness and optimize treatment matching, improving outcomes beyond one-size-fits-all approaches

## Limitations

These insights underscore the critical importance of investigating obesity from its earliest preclinical manifestations by identifying physiological markers of body resilience through examination of white and brown adipose tissue dynamics. While distinguishing between brown and white fat remains clinically challenging, recent advances in predictive modeling that assess physiological resilience offer unprecedented opportunities for early diagnosis and prevention of metabolic alterations. Thus, predicting obesity and identifying its subtypes through this approach should be central to the complexity of subtype-specific diagnostic and treatment algorithms. Implementing the adipocentric model with resilience assessment for obesity management in modern healthcare systems requires collaboration among researchers, educators in medical sciences, policymakers, and industry, and medical education to enact changes in clinical practice and ensure personalized medicine. Multidisciplinary clinician trainings, as well as patient education based on understanding individual physiological resilience, engagement with personalized but potentially more demanding interventions (e.g., graduated cold exposure, specialized exercise regimens), remains a limitation.
