# Supplementary material for: What role will physiological resilience play in brown-white fat dynamic in obesity management?
Source: Front Pharmacol. 2026 Jan 5;16:1691149. doi: 10.3389/fphar.2025.1691149 (PMC12812910; doi:10.3389/fphar.2025.1691149)
Supplement: Supplementary file 1 [file Supplementaryfile1.docx]

***Supplement 1.* Specific examples of obesity subtype differentiation regarding clinical obesity criteria.**

*Metabolically healthy obesity* (MHO) versus *metabolically unhealthy obesity* (MUO).

The adipocentric approach distinguishes individuals with similar BMI but divergent metabolic profiles. MHO presents *with preserved insulin sensitivity, standard lipid profiles, and lower visceral adipose tissue, despite elevated total body fat*, whereas MUO demonstrates *insulin resistance, dyslipidemia, and increased visceral fat accumulation, accompanied by elevated pro-inflammatory adipokines* (TNF-α, IL-6) [].

*Sarcopenic Obesity versus Non-Sarcopenic Obesity:*

This critical distinction identifies individuals with concurrent muscle mass depletion and excess adiposity, detectable through BIA or DXA assessment of appendicular lean mass, who require protein-enriched interventions and resistance training rather than caloric restriction alone, which could exacerbate muscle loss [].

*TOFI (Thin Outside, Fat Inside) phenotype versus* *classical obesity*:

The adipocentric model identifies individuals with a normal BMI (<25 kg/m²) but with excessive visceral and ectopic fat deposition in the liver, pancreas, and skeletal muscle, which can be detected through CT/MRI or elevated liver enzymes. This suggests metabolic-associated fatty liver disease (MAFLD), and these individuals face metabolic risks like those with clinical obesity despite having a normal appearance.

*Android (apple-shaped) versus gynoid (pear-shaped) obesity*:

WHR differentiation (>0.9 for men; >0.85 for women in the android pattern) identifies upper body obesity associated with higher cardiometabolic risk, increased cortisol reactivity, and reduced brown fat activity, which require cold exposure therapy and stress management, compared to lower body obesity that has relatively preserved metabolic health and different therapeutic priorities [].

*Brown adipose tissue deficit obesity:*

This diagnostic approach for brown-white fat dynamics helps address individual physiological resilience profiles to achieve personalized functionality, rather than relying on universal therapeutic interventions. Moreover, the adipocentric framework identifies individuals with impaired cold-induced thermogenesis and reduced brown fat activity (assessable through cold tolerance testing or thermal imaging), who benefit specifically from graduated cold exposure protocols (15-18°C for 15 mins until 90 mins daily) and dietary thermogenic compounds (capsaicin, green tea catechins) to restore metabolic flexibility.

This precision subtyping enables personalized therapeutic algorithms.

**Supplement 2. Standardized Cold Challenge Protocol**

To assess brown fat thermogenic capacity and ANS responses under controlled conditions, a standardized cold exposure testing protocol should incorporate:

**Pre-Test requirements:** Participants must complete a specified fasting duration, adhere to medication restrictions, and undergo ambient temperature acclimation to establish baseline physiological parameters.

**Cold exposure parameters:** Subjects are exposed to an environmental temperature of 15-18°C for 15-90 minutes while wearing standardized clothing to ensure consistent thermal challenge across participants.

**Concurrent physiological measurements:** Infrared thermography is performed at 15-minute intervals to map brown fat activation, heart rate variability (HRV) is continuously monitored to assess ANS function, and blood glucose is sampled hourly to track metabolic responses.

**Safety monitoring:** Predetermined termination criteria are applied to protect participant throughout the exposure period.

**Supplement 3. 5-Steps Process for Brown-White Fat Dynamics Assessment**

- Step 1: Advanced anthropometric screening and ANS flexibility profiling by HPV
- Step 2: Clinical biomarker assessment (glycemic and lipidic profiles, inflammatory markers, liver enzymes)
- Step 3: Advanced instrumentation* (BIA, DXA, CT/MRI)
- Step 4: Subtype assignment (MHO, MUO, Sarcopenic Obesity, TOFI, Pathologically Obese)
- Step 5: BAT Activity & Therapeutic Intervention

*Detailed specifications for each modality in the multimodal digital biomarker assessment system, including: (1) Near-infrared spectroscopy protocols for tissue oxygenation measurement, (2) Infrared thermography standardized cold challenge procedures and supraclavicular region-of-interest definitions, (3) Bioelectrical impedance analysis (BIA) frequency ranges and phase angle interpretation, (4) Core-to-peripheral temperature differential measurement protocols with standardized environmental conditions.
